# The Effectiveness and Safety of Thunder Fire Moxibustion for Treating Allergic Rhinitis: A PRISMA Compliant Systematic Review and Meta-Analysis

**DOI:** 10.1155/2020/6760436

**Published:** 2020-09-19

**Authors:** Ting Yuan, Jun Xiong, Jun Yang, Xue Wang, Yunfeng Jiang, Xiaohong Zhou, Kai Liao, Lingling Xu

**Affiliations:** ^1^Institute of Acupuncture, Moxibustion and Tuina, Jiangxi University of Traditional Chinese Medicine, Nanchang, Jiangxi, China; ^2^Department of Acupuncture and Moxibustion, The Affiliated Hospital of Jiangxi University of TCM, Nanchang, Jiangxi, China

## Abstract

**Background:**

Allergic rhinitis (AR) is a noninfectious inflammatory disease caused by allergic individuals exposed to allergens. Western medicine therapy for treating AR causes obvious adverse events, while thunder fire moxibustion (TFM) is known as a safe and effective treatment for AR. Therefore, we conducted this meta-analysis to evaluate the effectiveness and safety of TFM for treating AR.

**Methods:**

PubMed, Web of Science, Embase, the Cochrane Library, CNKI, WanFang, VIP, and CBM from inception to April 5, 2020, were searched without any language restriction. Reviewers identified studies, extracted data, and assessed the quality, independently. The primary outcomes were the total effective rate and the TNSS. The secondary outcomes included TNNSS, RQLQ, VAS, serum IgE, IgA, or IgG level, and adverse events. Randomized controlled trials (RCTs) were collected; methodological quality was evaluated using the Cochrane risk of bias assessment tool (RoB), and the level of evidence was rated using the GRADE approach. Meta-analysis was performed using the RevMan5.3.0 software.

**Results:**

A total of 18 RCTs were included, including 1600 patients. The results of this meta-analysis showed a statistically significant effect in a total effective rate of T = TFM (RR = 1.07; 95% CI [1.03, 1.12]; *P* = 0.45; *I*^2^ = 0%) and T = TFM + other treatments (RR = 1.18; 95% CI [1.11, 1.25]; *P* = 0.03; *I*^2^ = 53%). In addition, TFM intervention also showed significant difference in total symptom score (T = TFM + other treatments) (MD = −1.42; 95% CI [−1.55, −1.29]; *P* = 0.03; *I*^2^ = 60%) in patients with AR.

**Conclusion:**

Existing evidence shows that TFM is safe and effective for AR. Due to the universal low quality of the eligible trials and low evidence level, we should draw our conclusions with caution. Therefore, clinical researchers should carry out more large-sample, multicentre, high-quality randomized controlled clinical trials in the future to verify the clinical efficacy of TFM in treating AR.

## 1. Introduction

Allergic rhinitis (AR) is a serious global health problem affecting people of all ages in most countries of the world. It is a chronic nasal inflammation caused by exposure of allergic individuals to allergens and can occur within minutes of exposure [1]. Among the allergens of AR, those who are allergic to cold air, pollen, dust and mites are more common. Its main pathological feature is allergen-specific immunoglobulin E (IgE), which binds to the IgE receptors on mast cells and basophils, releases chemical mediators such as histamine, leukotrienes, and cytokines, and acts on nasal mucosa that can lead to the development of allergic rhinitis symptoms [2]. Hyperfunction of T-helper-type 2 mucosal cells accelerates the process of AR [3]. The main symptoms of AR include paroxysmal sneezing, watery nose, nasal itching, and nasal blockage. Besides, some patients also have ocular symptoms, such as itchy eyes, red eyes, and tears [4]. The clinical diagnosis of AR is based on detailed medical history, symptoms, signs, positive skin prick test (SPT), and specific serum IgE levels. At the same time, AR can be classified as light, medium, or severe grades according to its severity [5]. The severity and recurrence of allergic rhinitis seriously affect the quality of life of the patients, including the decrease in working and learning ability and the disorders of sleep and mood [6]. In recent years, the incidence of AR has been on a sharp rise, currently affecting about 10–20% of the population worldwide [7]. Epidemiological surveys show that the prevalence of AR varies from country to country around the world. In American adults, the prevalence of AR ranges from 10 to 30% [8, 9]; in Peru, the overall prevalence of allergic rhinitis was 18%; and in the mainland of China, the prevalence of AR is 4–38% [10].

Currently, the treatment methods for AR are mainly western medicines [11]. Among them, commonly used western medicines include loratadine tablets and inhaled budesonide, and so on [12]. However, the medicines have significant side effects, such as drowsiness, dryness of mouth and nose, and epistaxis. Therefore, the medicines should not be taken for a long time [13].

Thunder fire moxibustion (TFM) is a traditional Chinese medicine (TCM) treatment. It is made of moxa sticks with traditional Chinese medicine powder and moxa wool. It is like a big firecracker. After lighting, ten layers of cotton paper were placed on the acupuncture points to press and warm moxibustion. It produces heat to stimulate specific acupuncture points or parts of the body surface, regulates the function of visceral organs by stimulating meridian qi with the help of thermal radiation, and improves the circulation by penetrating deep tissue through heat [14].

Although several clinical trials have been conducted on TFM for treating AR, no systematic review and meta-analysis of TFM or TFM combined with other treatments for treating AR are yet reported. Intriguingly, many high-quality clinical trials have reported that western medicine has severe adverse reactions, and long-term use is prone to drug resistance. Still, TFM has fewer adverse reactions and higher safety. Hence, the goal of this study was to evaluate the quality of these RCTs to assess the effectiveness and safety of TFM in treating AR and guide clinicians better.

## 2. Methods

### 2.1. Protocol and Registration

We conducted a systematic review and meta-analysis in strict accordance with PRISMA (The Preferred Reporting Items for Systematic Review and Meta-analysis) statement [15]. The PRISMA checklist was presented in an online supplementary appendix 1. The protocol was beforehand registered in PROSPERO 2019 CRD42019141113. And it could be found from http://www.crd.york.ac.uk/PROSPERO/display_record.php?ID=CRD42019141113.

### 2.2. Inclusion Criteria

#### 2.2.1. Types of Studies

All relevant randomized controlled trials (RCTs) or quasi-RCTs of TFM for AR were collected.

#### 2.2.2. Participants

Participants diagnosed with AR according to the allergic rhinitis and its impact on asthma (ARIA) [16] were included. No limitation was set on the patients' age, gender, occupation, ethnic group, disease duration, syndrome type, source of cases, or disease severity.

#### 2.2.3. Types of Interventions and Comparators

For the trial group, TFM alone or TFM combined with other positive interventions (e.g., western medicine and conventional therapy) were eligible. Excluded therapies were the RCTs of TFM not as a primary therapy. For the control group, positive comparators (e.g., western medicine and conventional therapy), no treatment, and placebo or sham TFM were eligible.

#### 2.2.4. Types of Outcome Measures

Primary outcomes were the total effective rate and the total nasal symptom score (TNSS) [17], which was recorded from a validated daily or weekly diaries or visual analog scale (VAS). The TNSS consisted of four nasal symptoms (rhinorrhea, nasal itching, nasal obstruction, and sneezing) using a five-point scale from 0 to 4 (0 = no symptom, 1 = mild, 2 = moderate, 3 = severe, and 4 = very severe). The TNSS was obtained from the sum of all four individual symptom scores, with a total possible score ranging from 0 (no symptoms) to 16 (maximum symptom intensity). Secondary outcomes of interest were presented as follows: (1) total nonnasal symptom score (TNNSS) [17]; (2) rhinitis quality of life questionnaire (RQLQ) [18]; (3) VAS (visual analog scale); (4) laboratory indicators: serum IgE, IgA, or IgG levels; and (5) adverse events.

### 2.3. Electronic Search Methods

PubMed, Web of Science, Embase, the Cochrane Library, China National Knowledge Infrastructure (CNKI), WanFang, VIP, and CBM from the inception to April 5, 2020, were searched without any language restriction, but involving only the human subjects. The main keywords included “thunder fire moxibustion,” “allergic rhinitis,” and “RCT.” Also, the searches were rerun before the final analysis that followed the PRISMA checklist. Besides, the grey literature and the references of all included literatures were retrieved manually. The full-search strategy for PubMed is shown in Table 1.

### 2.4. Selection Process

Three independent researchers (TY, WX, and JY) selected qualified literature strictly according to Cochrane Collaborative System Evaluator's Handbook 5.2.0. [19]. Disagreement was resolved by a tripartite discussion or the fourth researcher (JX).

### 2.5. Data Extraction and Management

Based on the PICOS principle, we set up the standard data extraction table in advance. Before the formal data extraction, preextraction was conducted twice to ensure the smooth progress of the formal extraction. Data extraction was carried out independently by three researchers (TY, WX, and JY) and cross-checked repeatedly. Disagreement was resolved by a tripartite discussion or the fourth researcher (JX). Meanwhile, the intention-to-treat (ITT) analysis was applied to the missing data. Excel 2007 was used for data extraction. Relevant contents of data extraction included title, author, publication time, average age, sample size, disease type, course of treatment, intervention measures, control measures, adverse reactions, and outcome indicators. When essential data in the literature was missing or incomplete, the study author was contacted by phone or e-mail.

### 2.6. Assessment of the Methodological Quality

We evaluated the methodological quality of qualified RCTs using the Cochrane risk assessment tool [20] according to Cochrane Reviewer's Handbook 5.0. It contains seven items: random sequence generation, allocation concealment, blinding of participants or doctors, blinding of outcome evaluator, incomplete outcome data, selective outcome reporting, and other biases. High (H), low (L), and unclear (U) were used to evaluate the degree of risk of bias in each item. Three reviewers (TY, WX, and JY) cross-checked the evaluation results of the included study, respectively. Disagreement was resolved by a tripartite discussion or the fourth researcher (JX).

### 2.7. Data Synthesis and Analysis

Meta-analysis was performed using RevMan5.3.0 software. The data were summarized using risk ratios (RRs) with 95% CI for binary outcomes or mean difference (MD) with 95% CI for continuous outcomes. Chi-square test and *I*^2^ value were used to test the degree of heterogeneity. When *P* < 0.1, *I*^*2*^ > 50%, no heterogeneity was considered among the trials, and the fixed effect model was used for statistical analysis; otherwise, the random effect model was used. When the clinical heterogeneity between the two studies was substantial, only descriptive analysis was performed. The potential publication bias was tested by using an inverted funnel chart developed by Egger when the number of qualified RCTs was more than 10 [21]. Also, we conducted subgroup analysis and sensitivity analysis to explore the source of heterogeneity.

### 2.8. Level of Evidence

We selected the Grading of Recommendations, Assessment, Development and Evaluation (GRADE) system to evaluate the level of evidence quality [22]. RCTs started with high level of evidence. We lowered the level of evidence (high, moderate, low, and very low) gradually from the five aspects, including risk of bias, inconsistency, imprecision, indirectness, and publication bias.

## 3. Results

### 3.1. Search Results

**Figure 1:**
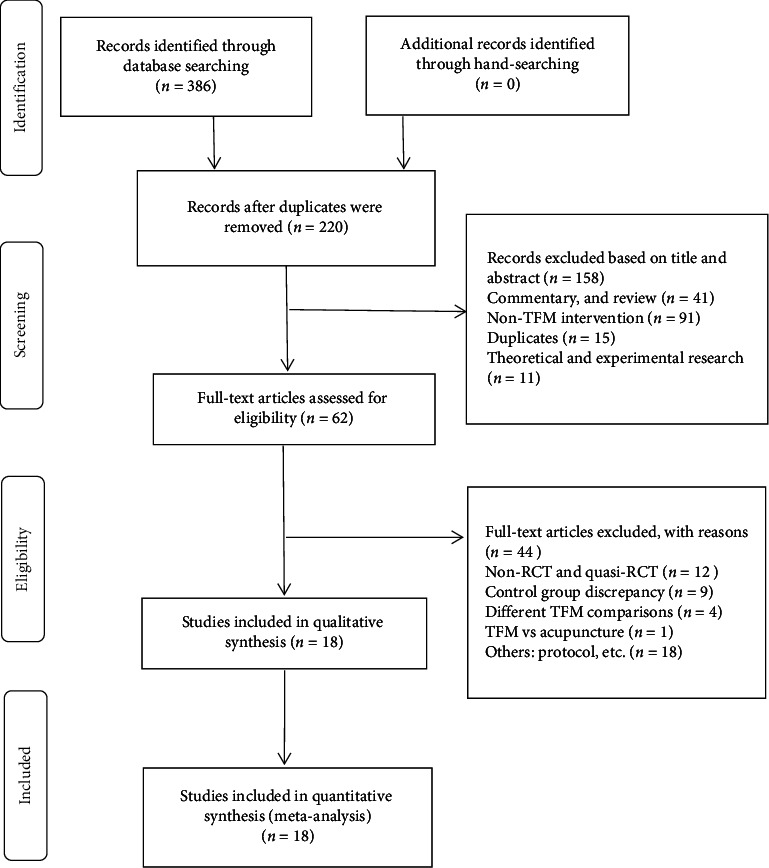
Flowchart of literature selection.

Three hundred eighty-six literatures were initially retrieved: 20 from PubMed, 24 from Web of Science, 36 from Embase, 11 from Cochrane Library, 63 from CNKI, 49 from WanFang, 48 from VIP, and 135 from CBM. NoteExpress 3.0 software was used to classify and screen the initial study and eliminate the reviews that did not meet the inclusion criteria. Finally, a total of 18 RCTs were included (Figure 1).

### 3.2. Study Characteristics

Record all characteristics of the included trials. All the studies were published between 2005 and 2019. There were 856 cases in the treatment group and 854 cases in the control group. There were six trials, including three control groups, but only two of them met the criteria. So we only extracted baseline data for these two groups. The number of patients in each clinical study ranged from 22 to 103. Most patients were recruited from the outpatient or inpatient departments. Besides, the result data and other information for each included study were presented in Table 2.

#### 3.2.1. Types of Studies

The eligible studies included 14 randomized controlled trials (RCTs) and 4 quasirandomized controlled trials (quasi-RCTs).

#### 3.2.2. Types of Intervention

7 RCTs [23–29] adopted TFM treatment alone, 1 RCT [30] adopted TFM + budesonide nasal spray treatment, 1 RCT [31] adopted TFM + TCM treatment, 1 RCT [32] adopted TFM + tuina treatment, 1 RCT [35] adopted TFM + acupoint patching treatment, and 7 RCTs [33, 34, 36–40] adopted TFM + acupuncture treatment.

#### 3.2.3. Types of Control

7 RCTs [23–28, 30] adopted western medicine treatment, 1 RCT [29] adopted no treatment, 1 RCT [31] adopted traditional Chinese medicine treatment, and 9 RCTs [32–40] adopted acupuncture and moxibustion treatment.

#### 3.2.4. Types of Outcome Measures

15 RCTs [23–28, 30, 31, 33–38, 40] assessed the total effective rate, 9 RCTs [23, 28, 32–35, 37–39] selected symptom score, 1 RCT [23] assessed VAS score, 1 RCT [23] assessed RQLQ, and 1 RCT [29] assessed the serum of IgE and IgG levels, respectively.

### 3.3. Risk of Bias Assessment

**Figure 2:**
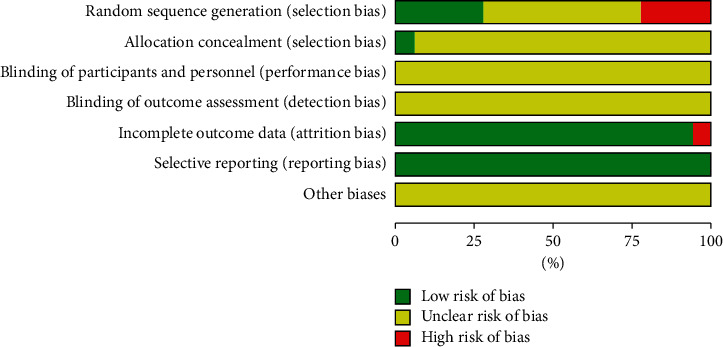
Risk of bias graph.

**Figure 3:**
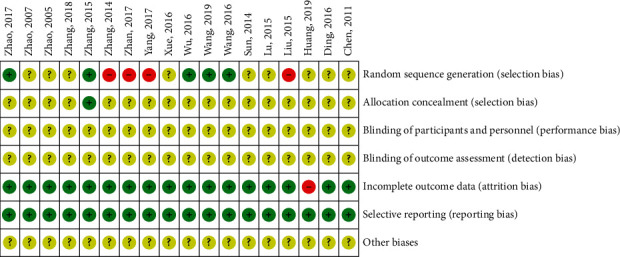
Risk of bias summary.

(1) Randomization: 2 RCTs [23, 31] were randomized by random number table, 2 RCTs [24, 36] were randomized by draw, 1 RCT [25] was randomized by computer, 2 RCTs [34, 35] were randomized by odd-even order, 2 RCTs [38, 40] were randomized by registration order, and 9 RCTs [26–30, 32, 33, 37, 39] were randomized word only; (2) allocation hiding: only 1 RCT [25] mentioned proper allocation hiding, and the remaining 17 RCTs did not mention whether allocation hiding; (3) blind method: none of the trials mentioned the blind method; (4) selective report: all studies reported preset outcome indicators; and (5) follow-up and abscission: only 1 RCT [32] did not report the causes of cases of abscission in detail, so rated as high risk, as shown in Table 3 and Figures 2 and 3.

### 3.4. Outcomes

#### 3.4.1. Total Effective Rate

**Figure 4:**
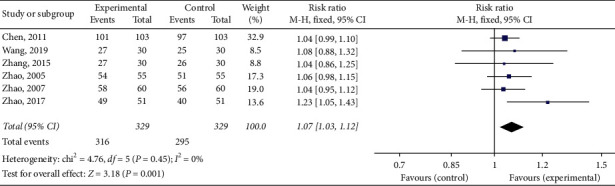
Forest plots of total effective rate (trial group = TFM).

**Figure 5:**
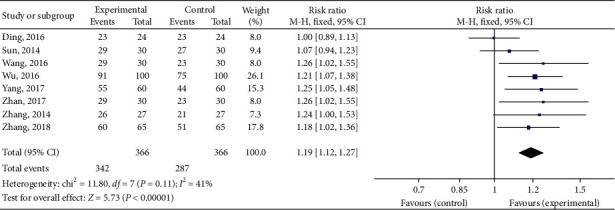
Forest plots of total effective rate (trial group = TFM + other treatments).

The total effective rate was reported in 15 [23–28, 30, 31, 33–38, 40] out of 18 studies. The total effective rate of trial group = TFM was reported in 6 studies [23–28] and that of trial group = TFM + other treatments was reported in 9 studies [30, 31, 33–38, 40]. The total effective rate (trial group = TFM) had statistical significance (RR = 1.07; 95% CI [1.03, 1.12]; *P*=0.45; *I*^2^ = 0%) with low or no heterogeneity (Figure 4). The total effective rate (trial group = TFM + other treatments) had statistical significance (RR = 1.18; 95% CI [1.11, 1.25]; *P* = 0.03; *I*^2^ = 53%) with higher heterogeneity (Figure 5). The result showed that TFM had a better effect compared with the control group.

#### 3.4.2. Total Symptom Score

**Figure 6:**
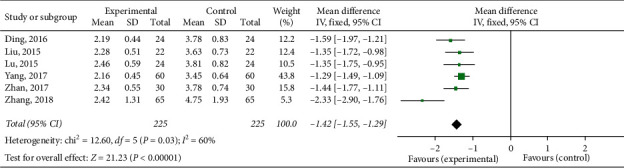
Forest plots of total symptom score (trial group = TFM + other treatments).

The total symptom score was reported in 9 [23, 28, 32–35, 37–39] out of 18 studies. Since two studies [23, 32] described only a single symptom integral and did not count the total symptom score, only 7 studies were included for meta-analysis. The total symptom score of trial group = TFM was reported in one study [28], and that of trial group = TFM + other treatments was reported in 6 studies [33–35, 37–39]. Only 1 RCT [28] was included in trial group = TFM, so descriptive analysis was conducted. And the results showed that the TFM had a significant effect on the clinical symptoms of AR patients, and it was better than the control group. The total symptom score (trial group = TFM + other treatments) had statistical significance (SMD = −1.42; 95% CI [−1.55, −1.29]; *P*=0.03; *I*^2^ = 60%) with higher heterogeneity (Figure 6). The result showed that TFM had a better effect compared with the control group.

#### 3.4.3. VAS Score

Only 1 RCT [23] was included, so descriptive analysis was conducted. And the results showed that the comparison of VAS scores between the two groups was statistically significant (*P* < 0.001). VAS score of the TFM group after treatment was lower than that of the western medicine group, indicating that, after treatment, VAS score of the TFM group could be reduced. Still, the change range was lower than that of the western medicine group.

#### 3.4.4. Rhinitis Quality of Life Questionnaire (RQLQ)

Only 1 RCT [23] was included, so descriptive analysis was conducted. And the results showed that the difference of RQLQ score between the TFM group and the western medicine group was statistically significant (*P* < 0.001). The RQLQ score of the TFM group after treatment was lower than that of the western medicine group, indicating that, after treatment, the TFM group could reduce the RQLQ score, but the reduction was lower than that of the western medicine group.

#### 3.4.5. Serum IgE and IgG Levels

Only 1 RCT [29] was included, so descriptive analysis was conducted. And the results showed that, compared with the blank control group, *P* > 0.05, indicating that no statistically significant difference in serum IgG between the healthy population and allergic rhinitis patients; *P* < 0.05, indicating that serum IgE was statistically significant between the healthy population and allergic rhinitis patients.

#### 3.4.6. Adverse Events

Of 19 trials, only 1 trial [23] reported on adverse events, which reported that no adverse events occurred. No adverse events were reported in the remaining 18 trials.

#### 3.4.7. TFM Performed for AR

We also analyzed the selection of acupoints for the included RCTs. A total of 17 acupoints were selected from 18 studies. Two studies [23, 26, 36, 40] selected the same acupoint therapy, respectively, and three other studies [27, 28, 30, 37–39] selected another same acupoint therapy, respectively, but the remaining studies were different. DU29/LI20 (16 studies [23–30, 33–40], 88.9%) had the highest frequency of use, followed by DU23/LI4 (13 studies [23–30, 33–40], 72.2%), DU25 (12 studies [23–28, 30, 34, 35, 37–39], 66.7%), BL1 (9 studies [23, 26–28, 30, 34, 37–39], 50.0%), LU7 (6 studies [23, 24, 26, 37–39], 33.3%), and BL13/EX-HN8 (3 studies [25, 32, 34, 36, 40], 16.7%).The other acupoints were used only one time, as shown in Table 4.

#### 3.4.8. Publication Bias

**Figure 7:**
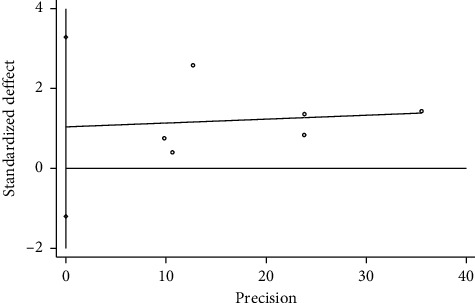
Regression diagram of Egger's test based on total effective rate (T = TFM).

**Figure 8:**
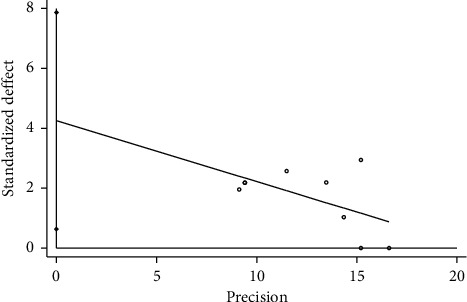
Regression diagram of Egger's test based on total effective rate (T = TFM + other treatments).

Based on the total effective rate (trial group = TFM) of STATA 12.0 software, we analyzed publication bias through Egger's test, and the results showed that *P* = 0.267 > 0.05, and the 95% CI [−1.201, 3.285] contained 0, suggesting that the possibility of publication bias was small. Based on the total effective rate (trial group = TFM + other treatments), the results showed that *P* = 0.027 < 0.05, and the 95% CI [0.63, 7.86] did not contain 0, suggesting that the possibility of publication bias was bigger, as shown in Figures 7 and 8.

#### 3.4.9. Subgroup Analyses

Because there was no enough data, we did not conduct a subgroup analysis for different groups.

#### 3.4.10. Sensitivity Analysis

**Figure 9:**
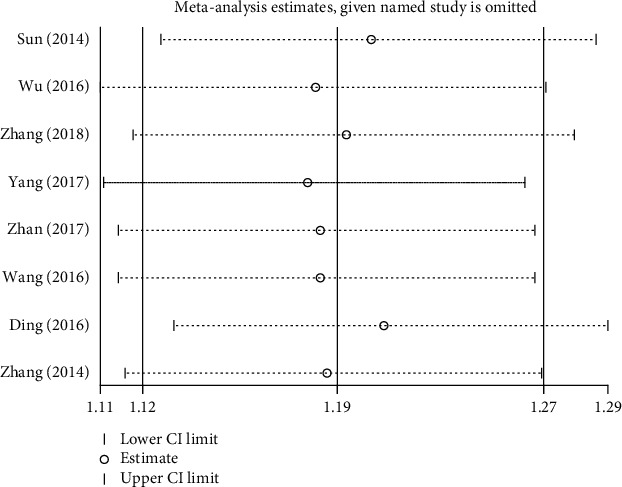
Sensitivity analysis plot of the total effective rate.

**Figure 10:**
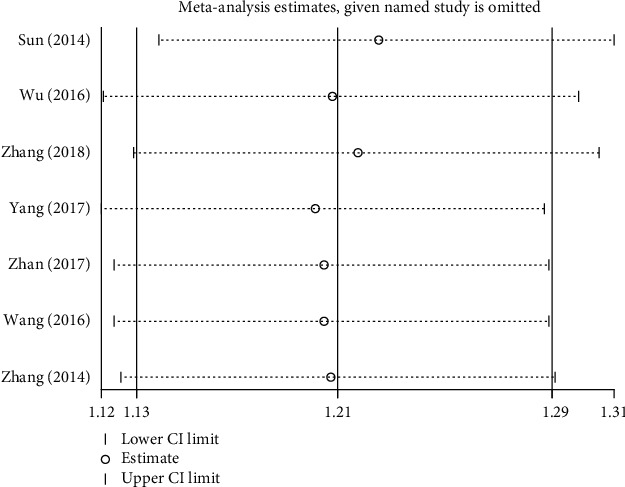
Sensitivity analysis plot of the total effective rate after omitting the Ding study.

Sensitivity analysis was used to evaluate the stability of meta-analysis. We performed a sensitivity analysis by using STATA 12.0 software, such as the effective rate. Sensitivity analysis showed that the results of the effective rate were not stable. We found that the results of heterogeneity comparing the effective rate were significantly reduced (RR = 1.19, 95% CI = 1.12 to 1.27, *P* = 0.161, *I*^2^ = 33.4%) by omitting the study by Ding conducted in 2016 [37]. Therefore, this study [37] was considered as the source of heterogeneity, as shown in Figures 9 and 10.

### 3.5. Level of Evidence

The results of GRADE analysis showed that the evidence quality of all outcome indicators was low or very low, which was not conducive to our recommendation of the results. We reduced the levels mainly by the risk of bias, inconsistency, and imprecision, as shown in Table 5.

## 4. Discussion

### 4.1. Main Findings of TFM Intervention Effects

The results of this meta-analysis showed a statistically significant effect in total effective rate of trial group = TFM (RR = 1.07; 95% CI [1.03, 1.12]; *P* = 0.45; *I*^2^ = 0%) and trial group = TFM + other treatments (RR = 1.18; 95% CI [1.11, 1.25]; *P*=0.03; *I*^2^ = 53%). In addition, TFM intervention also showed significant differences in the total symptom score (trial group = TFM + other treatments) (MD = −1.42; 95% CI [−1.55, −1.29]; *P*=0.03; *I*^2^ = 60%) in patients with AR. Although the preset outcome indicators of VAS score, RQLQ score, and serum IgE and IgG levels were included in this study, only one RCT was included, and then, only descriptive analysis was conducted. And the results showed that the score of the treatment group was lower than the control group. Besides, TFM intervention has fewer adverse reactions. Therefore, TFM treatment for AR is safe and effective, worthy of clinical application.

### 4.2. Quality and Level of Evidence

The Cochrane risk of bias assessment showed that the quality of evidence in this study varied from low to moderate. Among them, only 1 case was of medium quality, and the remaining 17 cases were of low quality. So the quality of the included RCTs was generally low. Inappropriate random method, allocation concealment, and a lack of blinding of all studies exaggerated the results of the outcome measures. In this review, only 27.78% of the studies and 5.56% of the studies reported correct randomization and allocation of concealment, respectively, which can result in overestimation. Due to the low level of evidence, we recommended TFM to treat AR finitely.

### 4.3. Discussion of Heterogeneity

The total effective rate of TFM for treating AR showed apparent heterogeneity. To find the source of heterogeneity, we conducted sensitive analysis and found that, after excluding the study by Ding conducted in 2016 [37], the results of heterogeneity comparing the effective rate was obviously reduced (RR = 1.19, 95% CI = 1.12 to 1.27, *P* = 0.161, *I*^2^ = 33.4%). To trace its causes, we found that it had problems of low quality and small sample size. This suggests that the results of this meta-analysis were to some extent influenced by the risk of bias.

### 4.4. Limitations and Advantages

The present study presented several limitations, as follows.

Firstly, although we collected the abundant literature without any language restriction through a comprehensive searching strategy of nine different databases, we could not be sure that all relevant RCTs were included.

Secondly, limited by the retrieval conditions, only the Chinese and English databases were searched, rendering some language biases. All included studies were published in Chinese and none in English, which restricts the generalizability of the findings due to the context in terms of tradition and culture.

Thirdly, the methodological quality of most eligible trials was low, and there was a severe risk of bias, which reduced the authenticity and reliability of TFM evidence for AR in this study. Although the “random word” was used in 14 of the 18 studies, only 5 correctly described the random method. At the same time, almost all eligible studies did not implement allocation concealment and blind method, which may result in severe implementation of bias and selective bias.

Finally, most of the meta-analysis in this review showed high heterogeneity. And due to the small number of studies included in some outcome indicators and the lack of data in some studies, the subgroup analysis was not carried out according to the preset possible variables.

The study also presented some glaring advantages, as follows.

Most importantly, there are no systematic reviews and meta-analysis of TFM for the treatment of AR. This is the first systematic review designed to evaluate the effectiveness and safety of TFM for AR patients. Finally, we strictly followed the PRISMA guideline for this systematic review and meta-analysis, and the content met the standards. Thus, we speculated that the results of this review could provide evidence on the efficiency and safety of TFM in treating AR, which would benefit the patients and practitioners.

## 5. Conclusion

This is the first systematic review and meta-analysis designed to assess the effectiveness and safety of TFM for AR patients. And this review included a comprehensive assessment of methodological quality and the level of evidence. Existing evidence shows that TFM is safe and effective in the treatment of AR. Due to the universally low-quality eligible trials and low evidence level, we should draw our conclusions with caution. And clinical researchers should carry out more large-sample, multicentre, high-quality randomized controlled clinical trials in the future to verify the clinical efficacy of TFM in treating AR.

## Figures and Tables

**Table 1:** PubMed: searched on April 5, 2020.

Search	Query
#1	“Allergic rhinitis” [Ti/Ab] or “rhinallergosis” [Ti/Ab] or “hypersensitive rhinitis” [Ti/Ab] or “anaphylactic rhinitis” [Ti/Ab] or “perennial rhinitis” [Ti/Ab] or “pollinosis” [Ti/Ab] or “seasonal allergic rhinitis” [Ti/Ab] or “nasal allergy” [Ti/Ab]
#2	“Thunder fire moxibustion” [Ti/Ab] or “thunder-fire needle” [Ti/Ab] or “thunder fire God moxibustion” [Ti/Ab]
#3	“Randomized controlled trial” [Ti/Ab] or “clinical trial” [Ti/Ab] or “randomized” [Ti/Ab]
#4	“Allergic rhinitis” [MeSH] or “Rhinitis, Allergic, Seasonal” [Mesh]
#5	“Moxibustion” [MeSH]
#6	“Randomized controlled trial” [MeSH] or “controlled clinical trial” [MeSH]
#7	#1 OR #4
#8	#2 OR #5
#9	#3 OR #6
#10	#7 AND #8 AND #9

**Table 2:** Basic characteristics of eligible RCTs.

Study ID	Sample size T/C	Age	Intervention		Period (w)	Adverse events	Outcome	Drop out	Moxibustion acupoint
			Trial group	Control group					
Wang [23]	60 (30/30)	T: 32.87 ± 11.10 C: 32.80 ± 10.61	TFM	Mometasone furoate nasal spray	3/3	Non	Total effective rate, VAS, RQLQ, symptom score	Non	DU23, DU25, DU29, LI20, LU7, LI4, BL1
Zhao et al. [24]	102 (51/51)	T: 45.33 ± 1.39 C: 45.29 ± 1.35	TFM	Budesonide nasal spray	3/3	NR	Total effective rate	Non	DU23, DU25, DU29, LI20, LU7, LI4
Zhang [25]	60 (30/30)	T: 24.72 ± 7.43 C: 25.10 ± 8.14	TFM	Flixonase aqueous nasal spray	4/4	NR	Total effective rate	Non	DU23, DU25, DU29, LI20, LI4, EX-HN8, BL12, DU14, BL13
Chen [26]	206 (103/103)	T: 8–55 C: 10–52	TFM	Oxymetazoline	3/3	NR	Total effective rate	Non	DU23, DU25, DU29, LI20, LU7, LI4, BL1
Zhao and Zhang [27]	120 (60/60)	T: 8–63 C: 12–64	TFM	Beclomethasone dipropionate aerosol nasal spray	3/3	NR	Total effective rate	Non	DU23, DU25, DU29, LI20, BL1
Zhao et al. [28]	110 (55/55)	T/C: 47.1 ± 16.6 (11–80)	TFM	Beclomethasone dipropionate aerosol nasal spray	3/3	NR	Total effective rate, symptom score	Non	DU23, DU25, DU29, LI20, BL1
Xue et al. [29]	60 (30/30)	T/C: 32.12 ± 9.69 (18–55)	TFM	No treatment	3/3	NR	IgE, IgG	Non	DU23, DU29, LI20, LI4
Sun [30]	60 (30/30)	T/C: 12–58	TFM + budesonide nasal spray	Budesonide nasal spray	3/3	NR	Total effective rate	Non	DU23, DU25, DU29, LI20, BL1
Wu and Zhou [31]	200 (100/100)	T/C: 10–70	TFM + TCM	TCM	3/3	NR	Total effective rate	Non	
Huang et al. [32]	58 (30/28)	T: 19.76 ± 1.76 (18–25) C: 19.62 ± 1.36 (18–23)	TFM + tuina	Tuina	3/3	NR	Symptom score	T: 0 C: 2	DU14, BL13, BL43, BL20, BL23
Zhang [33]	130 (65/65)	T: 35.69 ± 21.92 C: 34.62 ± 23.57	TFM + acupuncture	Acupuncture	3/3	NR	Total effective rate, symptom score	Non	DU29, LI20, LI4, RN12, RN4, RN6
Yang [34]	120 (60/60)	T/C: 37.3 ± 7.3 (10–51)	TFM + acupuncture	Acupuncture	3/3	NR	Total effective rate, symptom score	Non	DU23, DU25, DU29, LI20, BL1, LI4, BL13
Zhan [35]	60 (30/30)	T: 9.3 ± 6.1 C: 9.1 ± 5.9	TFM + acupoint patching	Acupoint patching	2/2	NR	Total effective rate, symptom score	Non	DU23, DU25, DU29, LI20, LI4
Wang [36]	60 (30/30)	T: 46.25 ± 7.13 (15–68) C: 46.02 ± 7.11 (15–67)	TFM + acupuncture	Acupuncture	2/2	NR	Total effective rate	Non	DU29, LI20, LI4, EX-HN8
Ding and Chang [37]	48 (24/24)	T: 29.1 ± 3.8 (9–49) C: 27.8 ± 4.1 (8–46)	TFM + acupuncture	Acupuncture	4/4	NR	Total effective rate, symptom score	Non	DU23, DU25, DU29, BL1, LU7, LI20, LI4
Liu et al. [38]	44 (22/22)	NR	TFM + acupuncture	Acupuncture	4/4	NR	Total effective rate, symptom score	Non	DU23, DU25, DU29, BL1, LU7, LI20, LI4
Lu [39]	48 (24/24)	T: 45.9 ± 12.4 (17–60) C: 47.8 ± 13.1 (16–62)	TFM + acupuncture	Acupuncture	4/4	NR	Symptom score	Non	DU23, DU25, DU29, BL1, LU7, LI20, LI4
Zhang [40]	54 (27/27)	T: 15–68 C: 16– 66	TFM + acupuncture	Acupuncture	2/2	NR	Total effective rate	Non	DU29, LI20, LI4, EX-HN8	

*Note.* TFM = thunder fire moxibustion; TCM = traditional Chinese medicine; NR = not reported; VAS = visual analog scale.

**Table 3:** Risk of bias in the included RCTs.

Study	Random sequence generation	Allocation concealment	Blinding		Outcome data integrity	Selective outcome reporting	Other biases
			Patient/doctor blinding	Outcome assessor blinding			
Wang [23]	Random number table	Uncertain	Uncertain	Uncertain	Low risk	Low risk	Uncertain
Zhao et al. [24]	Draw random	Uncertain	Uncertain	Uncertain	Low risk	Low risk	Uncertain
Zhang [25]	Computer random	Low risk	Uncertain	Uncertain	Low risk	Low risk	Uncertain
Chen [26]	Random word	Uncertain	Uncertain	Uncertain	Low risk	Low risk	Uncertain
Zhao and Zhang [27]	Random word	Uncertain	Uncertain	Uncertain	Low risk	Low risk	Uncertain
Zhao et al. [28]	Random word	Uncertain	Uncertain	Uncertain	Low risk	Low risk	Uncertain
Xue et al. [29]	Random word	Uncertain	Uncertain	Uncertain	Low risk	Low risk	Uncertain
Sun [30]	Random word	Uncertain	Uncertain	Uncertain	Low risk	Low risk	Uncertain
Wu and Zhou [31]	Random number table	Uncertain	Uncertain	Uncertain	Low risk	Low risk	Uncertain
Huang et al. [32]	Random word	Uncertain	Uncertain	Uncertain	High risk	Low risk	Uncertain
Zhang [33]	Random word	Uncertain	Uncertain	Uncertain	Low risk	Low risk	Uncertain
Yang [34]	Random by odd-even order	Uncertain	Uncertain	Uncertain	Low risk	Low risk	Uncertain
Zhan [35]	Random by odd-even order	Uncertain	Uncertain	Uncertain	Low risk	Low risk	Uncertain
Wang [36]	Draw random	Uncertain	Uncertain	Uncertain	Low risk	Low risk	Uncertain
Ding and Chang [37]	Random word	Uncertain	Uncertain	Uncertain	Low risk	Low risk	Uncertain
Liu et al. [38]	Random by registration order	Uncertain	Uncertain	Uncertain	Low risk	Low risk	Uncertain
Lu [39]	Random word	Uncertain	Uncertain	Uncertain	Low risk	Low risk	Uncertain
Zhang [40]	Random by registration order	Uncertain	Uncertain	Uncertain	Low risk	Low risk	Uncertain

**Table 4:** The most frequently used acupoint.

Order	Acupoints	Frequency (%, *N* = 18)
1	DU29/LI20	16 (88.9%)
2	DU23/LI4	13 (72.2%)
3	DU25	12 (66.7%)
4	BL1	9 (50.0%)
5	LU7	6 (33.3%)
6	BL13/EX-HN8	3 (16.7%)
7	DU14	2 (11.1%)
8	BL12/BL43/BL2/BL23/RN12/RN4/RN6	1 (0.06%)

**Table 5:** Level of evidence.

Variable (study number)	Sample size (T/C)	*I * ^2^ value (%)	*P* value	Risk of bias	Inconsistency	Indirectness	Imprecision	Publication bias	Effect (95% CI)	Level of evidence
Total effective rate (T = TFM) (6)	359/359	0	0.046	Serious①	Non	Non	Serious③	Non	RR = 1.07, 95% CI = [1.03, 1.12]	Low⊕⊕○○
Total effective rate(T = TFM + other treatments) (9)	366/366	53.3	0.029	Serious①	Serious②	Non	Serious③	Serious④	RR = 1.18, 95% CI = [1.11, 1.25]	Very low⊕○○○
Total symptom score (9)	225/225	59.4	0.031	Serious①	Serious②	Non	Serious③	Non	RR = 1.20, 95% CI = [1.16, 1.23]	Very low⊕○○○

T: treatment group; C: control group. ①Blind method is missing, allocation hidden report is insufficient, and random method description is not clear; ②statistical heterogeneity and clinical heterogeneity were more significant; ③the total sample size was small, and OIS was not satisfied (optimal information size); ④Egger's test showed that *P* < 0.05, and the 95% CI [0.63, 7.86] did not contain 0, suggesting that the possibility of publication bias was bigger. ⊕⊕○○ represents the low level of evidence. ⊕○○○ represents the very low level of evidence.

## Data Availability

The data used to support the findings of this study are available from the corresponding author upon request.

## Abbreviations

AR: Allergic rhinitis
TFM: Thunder fire moxibustion
TNSS: Total nasal symptom score
TNNSS: Total nonnasal symptom score
RQLQ: Rhinitis quality of life questionnaire
VAS: Visual analog scale
ARIA: Allergic rhinitis and its impact on asthma
CI: Confidence interval
MD: Mean difference
RR: Risk ratio
RCT: Randomized controlled trial
PRISMA: The Preferred Reporting Items for Systematic Review and Meta-analysis
GRADE: Grading of Recommendations, Assessment, Development and Evaluation.
